# Identifying solution strategies in a mentalrotation test with gender-stereotyped objects

**DOI:** 10.16910/jemr.13.6.5

**Published:** 2021-03-10

**Authors:** Mirko Saunders, Claudia M. Quaiser-Pohl

**Affiliations:** University of Koblenz-Landau, Germany

**Keywords:** mental rotation, eye tracking, strategies, scan path, gender stereotype

## Abstract

Many studies deal with solution strategies in mental-rotation tests. The approaches range from global analysis, attention to object parts, holistic and piecemeal strategy to a combined strategy. Other studies do not speak of strategies, but of holistic or piecemeal processes or even of holistic or piecemeal rotation. The methodological approach used here is to identify mental-rotation strategies via gaze patterns derived from eye-tracking data when solving chronometric mental-rotation tasks with gender-stereotyped objects. The mental-rotation test consists of 3 male-stereotyped objects (locomotive, hammer, wrench) and 3 femalestereotyped objects (pram, hand mirror, brush) rotated at eight different angles. The sample consisted of 16 women and 10 men (age: M=21.58; SD=4.21). The results of a qualitative analysis with two individual objects (wrench and brush) showed four different gaze patterns. These gaze patterns appeared with different frequency in the two objects and correlated differently with performance and response time. The results indicate either an objectoriented or an egocentric mental-rotation strategy behind the gaze patterns. In general, a new methodological approach has been developed to identify mental-rotation strategies bottom-up which can also be used for other stimulus types.

## Introduction

Mental-rotation is the ability to rotate the mental representation of objects in mind and is a component of spatial object cognition [1]. The concept of mental rotation was first described and introduced into cognitive science by Shepard and Metzler [2]. Since the 1970s, various studies have been carried out; most of them concluded that male subjects in mental rotation outperform female subjects [e.g. 2, 3, 4].

However, the results appear to depend on the test used as a measuring instrument [4]. For example, the cube figures of mental rotation test (in the following MRT) are very similar to LEGO® bricks, dominoes, or other objects used in constructing and building toys, which are more commonly used by boys [5]. Rahe et al. [6] used male- and female-stereotyped objects for a paper and pencil mental rotation test. Generally, they found no sex differences for mental-rotation performance, whereas a significant interaction of sex and stimulus material revealed better performance for own-sex objects. It seems to be easier for subjects to solve mental- rotation tasks with objects they are more familiar with.

Another factor for the different performance in men and women in mental-rotation tasks appears to be the strategy used by the participants. The research literature distinguishes between holistic and analytic piecemeal strategies for mental-rotation tasks [e.g. 7, 8, 9]. Holistic strategies refer to mental transformations (e.g. mental rotation) of a stimulus as a whole, whereas analytic strategies involve comparisons of details of stimuli and reasoning processes [10]. This research refers e.g. to the work of Putz-Osterloh [11] and Putz-Osterloh and Lüer [12] who reported that subjects in the German Cube Comparison Test (CCT) [subtest of the German “Intelligence Structure Test” IST 3, 14] used different strategies to solve the items. For the IST-70 (Intelligence Structure Test), they identified cube tasks that can only be solved by surface strategies and tasks that require spatial reasoning for finding solutions. Gittler [15] made similar observations in the 3DW test. Scheer et al [9] in a pilot study with eye-tracking and EEG come to the assumption that men and women have different perception (visual search) and decision mechanisms, but similar mental rotation processes.

The holistic solution strategy is divided into an object-based and an egocentric approach [16]. In both approaches, the relationship between the intrinsic information of the object and the viewer is updated during mental rotation. Thus, in object-based rotation, the viewer's environment and egocentric reference system remain static, while the intrinsic coordinate system of the object is updated, whereas the egocentric approach updates the egocentric coordinate system in relation to the object's environment and intrinsic information.

In general, one challenge in this field of research is the identification of the strategies used. Often, the subjects are asked about their strategies after having been administered a mental-rotation test. Hence, in a questionnaire, selected answers are available and only a cross has to be made at the supposedly used strategy. Peters et al. [17] for example, asked whether the test persons rotated the illustrated figures completely or partially in their mind, whether they used movements such as fingers, hands or the pen to help themselves with the tasks. They also asked whether the test persons verbalized their thoughts in their minds.

The problem with this query of strategies is that they are only subjective assessments of a supposed strategy that do not have to match the real strategy. Other ways of identifying strategies have already been pursued. In a study by Janssen and Geiser [8] e.g., the relationship between solution strategies on the Mental Rotations Test [MRT; 17, 18] and the CCT [CCT 13, 14] was examined. These two tests are commonly used to identify different patterns of strategies. The researchers simultaneously analyzed the MRT and CCT item response patterns using latent transition analysis (LTA). The results showed that individuals using analytic (resp. holistic) strategies on the MRT tended to also use analytic (resp. holistic) strategies on the CCT [8]. The question is whether one can really grasp all possible strategies with this method, when only holistic and analytic strategies are assumed and searched quasi top-down. So far it is not exactly known which solution strategies for mental-rotation tasks exist at all. And this leads to the general question of whether it is possible to grasp strategies with a top-down method since, so far, only holistic (object-based and egocentric) and analytic strategies are referred to. as long as it is not known, which other strategies exist at all.

Other methods that deal with the duration of fixations and saccades only determine holistic and analytic strategies, too. Khooshabeh and Hegarty [19] or Nazareth et al. [20], for example, compared the number of successive fixations within each figure with the number of saccades between each figure. They postulate that when a holistic rotation strategy is used, the number of fixations within an object should be equal to the number of fixation saccades between objects. That is, during the holistic strategy, the participants look only once at the whole figure on each side to encode the whole figure, and then make a saccade to the other figure. However, if the participants used a piecewise strategy, they would make several fixations on a figure to look at different parts to rotate before making a saccade to the other figure.

Another problem with many eye-tracking studies is that none of the studies refers to the calculation of fixations and saccades. An eye-tracker records several points depending on the frequency, but these raw data must first be converted into fixations and saccades. Different algorithms are available for this purpose, depending on the software used. However, each algorithm and the frequency of the eye-tracker produces different fixations and saccades [see 21, 22, 23].

In this study, we followed a different, rather exploratory approach using an eye tracker to record the scan paths of subjects when solving a mental rotation task with gender-stereotyped objects. Thereby, we tried to analyze their gaze patterns in order to be able to identify solution strategies based on these patterns. Voyer et al. [24] criticizes this approach in other studies because they postulate that a paradigm of free viewing is usually applied, and the researchers attempted to determine a posteriori which fixation patterns might reflect a holistic and piecewise processing. However, this is not the approach of this study. We want to break away from the determined strategies without negating them and take a completely independent look at gaze patterns and possible strategies.

## Methods

### Participants

In order to check the suitability of this method, the first study consisted of a sample of (N=26) subjects, of which n=10 were men and n=16 were women. The participants in the study were aged 18 to 35 years, with the mean age being 21.58 years (SD = 4.21). All participants were students of the University of Koblenz-Landau, who participated in the study in the context of an empirical internship.

### Materials

**Figure 1:**
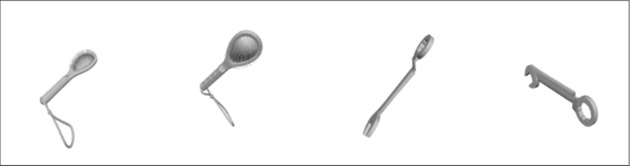
Examples of the stimuli; brush (left side) and wrench (right side) rotated in an angle of 90°. The initial object 0° is presented on the left and the rotated object on the right.

*Mental Rotation Test*. For the computerized Mental Rotation Test (cMRT) a self-created app was used, which was developed to represent rotated objects in different angles (stimulus Presentation). It is possible to select gender-stereotyped objects in the desired number and angles for the test, as well as to set a test run for the subjects. Furthermore, it is possible to give a feedback which is displayed in the form of a green or red square, depending on the correctly or incorrectly solved task, and occurring directly after the task has been solved. For this test, we used gender-stereotyped objects, which we developed by ourselves for former paper-and-pencil studies [25] (see Figure 1).

The computerized Mental Rotation Test (cMRT) consists of 3 male-stereotyped objects (locomotive, hammer, wrench) and 3 female-stereotyped objects (pram, hand mirror, brush). All stimuli, male- and female-stereotyped were displayed for all participants in pairs and in eight different angles (0°, 45°, 90°, 135°, 180°, 225°, 270°, 315°). The initial stimulus of each pairs was always shown on the left and at an initial angle of 0°, the comparison stimulus on the right. The comparison stimulus on the right side were presented either mirrored or not mirrored, rotated on the x-axis. Hence, a total of 96 different pairs were presented, showing 50% ‘mirrored and rotated on the x-axis’ and 50% ‘not mirrored and rotated on the x-axis’ pairs. Participants looked at a 19” monitor on which two three-dimensional gender-stereotyped figures (a pair) were presented and indicated whether the pair was the same but rotated, or different, i.e. mirrored and rotated. The time required by the test participants from the beginning of the exercise phase to the end of the test phase was between 14 - 36 minutes.

*Eye tracking measurement*. All eye movement metrics were captured with the screen based Tobii pro X3-120 Eye Tracker. For all recordings, we used a sampling rate of 120 Hz. Furthermore, a 5-point calibration of the eye tracker was performed before the experiment at each subject. A chin rest was not used during the experimental setup, because the eye-tracker tolerates a freedom of head movement of 30cm x 22cm x 30cm (width x height x depth) at a distance from the head to the monitor at 70 cm in 120 Hz mode with a maximum head movement speed of 35 cm/second. For recording of the eye movements, we used Tobii Studio©, a software for recording, analyzing and visualizing data from monitor-based eye trackers. The software supports the study process from data collection to interpretation and presentation of the results as well as the output of the data for further processing in Blickshift Analytics©.

### Procedure

Before the actual experiment was started, there was a practical phase with two gender-stereotyped items which were presented in all eight angles. After this practice phase, the actual test took place. Gender-stereotyped item pairs were also presented here. All 96 pairs were presented to the subjects randomly and continuously one after the other. The participants had to check whether it was a rotated or mirrored item and confirm their answer with the left or right arrow keys on the computer keyboard. A feedback sign in the form of a green or red box in the lower right corner was then displayed for one second. The next pair of items was immediately presented.

### Data Analysis

In order to test the suitability of the method itself, we have limited ourselves to the items wrench and brush. We chose these two items because the other items had relevant markers hidden in certain angular positions, so it was not always clear whether the item was rotated or mirrored. This is due to the fact that the other items are identically "drawn" from both sides. Therefore, the following results emerged from these two items.

The analysis of the eye-tracking data was done in different steps, which are described in the following subchapters.

*Analysis of scan paths*. One of the largest challenges in the analysis of eye-tracking experiments is the identification of similar viewing behavior. Here, the analysis software Blickshift Analytics© offers the possibility to recognize typical gaze sequences on area of interest (AOI) basis by an automatic procedure. Furthermore, it is possible to perform a direct search for exact gaze behavior and a similarity search [26].

*Analysis of fixations*. The first step to be able to perform an analysis of the gaze patterns is to prepare the raw data from the eye tracker in such a way that fixations can be identified. The Blickshift Analytics© software uses the dispersion-based algorithm I-DT to calculate raw data in fixations and saccades [22].

**Figure 2:**
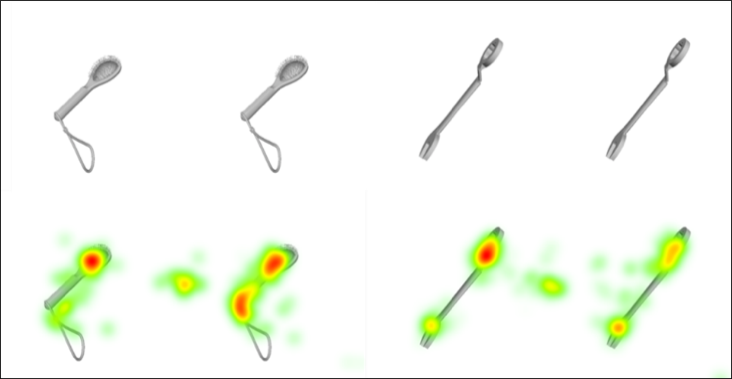
Brush and wrench at 0° angle above; below the corresponding heat maps

*Explorative definition of the AOIs*. The second step was to analyze and define the Areas of Interest exploratively. For this purpose, all fixations over all test persons of the respective stimuli were summarized with heat maps. The latter provide an initial overview of possible areas of interest. In Figure 2, the heat maps illustrate the areas of interest of every participant over the brush and wrench stimuli in a 0° angle. At the top, each pair is displayed as it appeared on the test person’s screen for better visibility. At the bottom is the pair with the overlaid heat maps. At this point, it can be stated that an accumulation of fixations occurs at certain prominent points. For example, in the case of the brush, it can be seen that the fixations at the brush head and at the transition from the handle to the strap accumulate. The fixation positions of the wrench are similar. In this context, too, the prominent points, the ends of the wrench, are considered as well. These areas are defined as areas of interest for the analysis. Once the areas of interest have been defined, the next step is to analyze the gaze patterns across these areas of interest.

*Sequence Analysis.* In order to determine similar or even identical gaze behavior in the test subjects, the gaze paths of each individual subject had to be analyzed optically. However, this method is not precise since several subjective factors influence the analysis. The Blickshift Analytics© software offers the solution to view parallel scan paths of all test persons and to analyze them by means of an AOI-based analysis function, patterns in eye movements and categorical data [see 27, 26].

*Explorative Sequence Search*. After the sequence analysis revealed patterns in the scan paths of the test subjects, we performed a sequence search on all test subjects based on the patterns found. This sequence search, which is performed in parallel for all test subjects, is suitable for finding given patterns in eye movements and categorical data (forward search) [see 27, 26].

## Results

**Figure 3:**
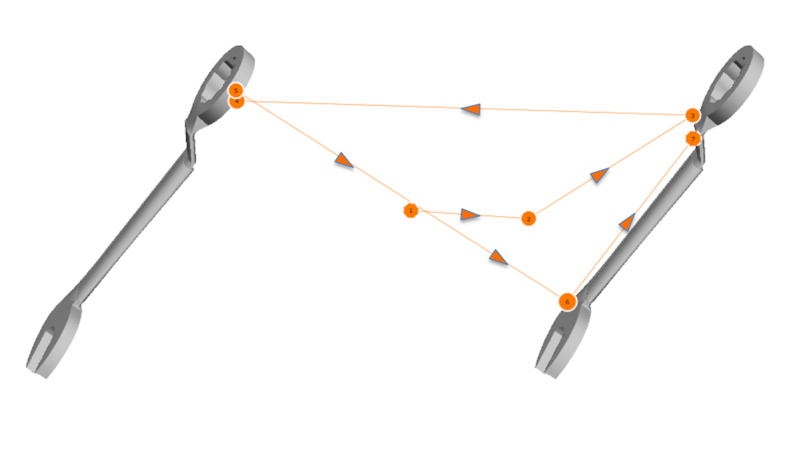
Gaze pattern 1 – Analytic

**Figure 4:**
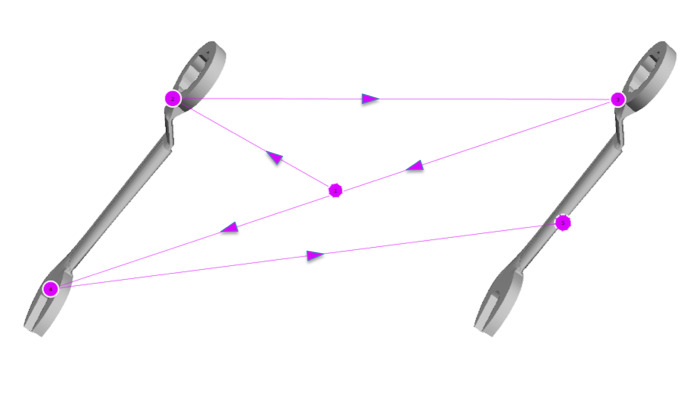
Gaze pattern 2 – Elaborate

**Figure 5:**
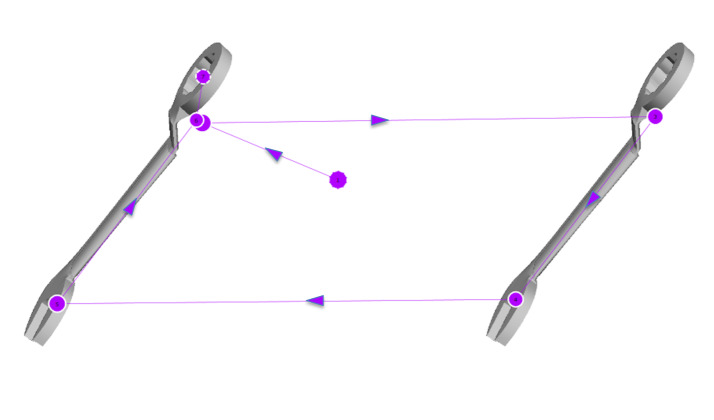
Gaze pattern 3 – Square

**Figure 6:**
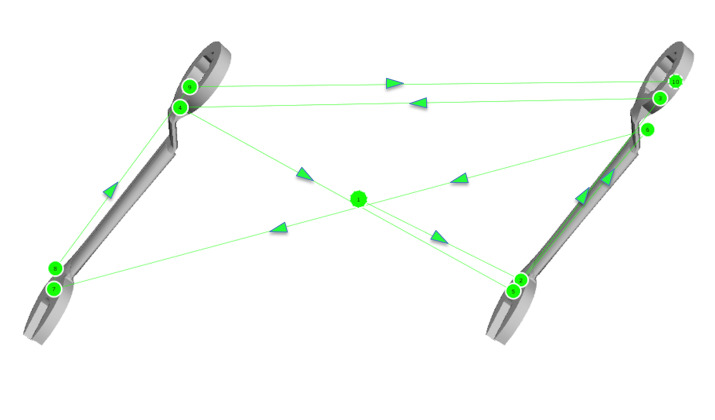
Gaze Pattern 4 - Uncertain

**Figure 7:**
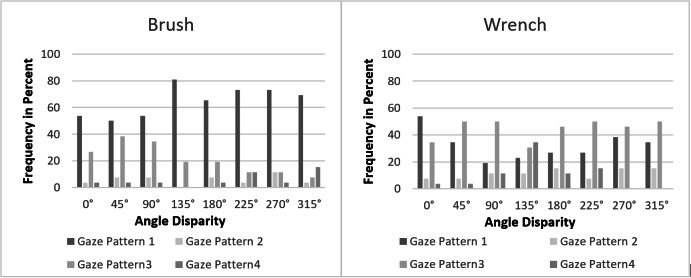
The frequency in percent of usage of the different gaze patterns separately for wrench and brush.

*Description of the gaze patterns.* As a result of the stepwise analysis described above, we found four different gaze patterns similarly for the two objects (wrench and brush). These gaze patterns are described in detail below. The following figures 3 to 6 show the initial items as well as the comparison items always at a 0° angle. This was chosen for clarity and uniformity. However, as can be seen in figure 7, all gaze patterns shown here occur at all angles.

*Gaze Pattern 1:* In the first gaze pattern (Figure 3), an essential feature of the object is identified and compared. The scan path often forms a triangle or runs only back and forth between the features to be compared. The viewing direction, i.e. from left to right first or from top to bottom, is irrelevant. One of the four AOI’s is completely excluded from the scan path and does not seem to be used to compare the stimuli at all. We called this gaze pattern ‘Analytic’. This term is not related to the analytic strategy, it rather goes beyond that and considers the way the test persons scanned the objects.

*Gaze Pattern 2:* In the second gaze pattern (Figure 4), all paired features of the stimuli are compared with each other from one side to the other. The scan path either forms a z-shape or a mirrored z-shape and looks as if one was reading a text. The starting point is again irrelevant. The direction in which the AOIs are scanned can be different. We called this gaze pattern ‘Elaborate’.

*Gaze Pattern 3:* In the third gaze pattern (Figure 5) all features are compared with each other. In contrast to the second gaze pattern, the view does not shift from one side to the other but from the second stimulus detail to the opposite direction. The scan path forms a square, which is the reason for calling this gaze pattern ‘Square’.

*Gaze Pattern 4:* In the fourth gaze pattern not, the similar features of the opposite object are considered but the different ones (Figure 6). This gaze pattern looks unstructured. Hence, this gaze pattern most frequently leads to incorrect answers. We called this gaze pattern ‘Uncertain’.

*Frequency of the gaze patterns for the items wrench and brush.* Figure 7 shows the frequencies of the four gaze patterns separately for the items wrench and brush and for the different angles. It can be seen that gaze pattern 1 and 3 were used most frequently for both items. Looking at the two items separately it can be noticed the for the wrench, a dominance of gaze pattern 3 can be found, which is used the most often for all angles. The second most frequently used gaze pattern with the wrench is gaze pattern 1, particularly used with the angles 0°, 45°, 270° and 315°. At the angle of 135°, however, the gaze patterns 1, 3 and 4 are almost equally represented with the wrench. For the brush, gaze pattern 1 is mainly used. Gaze pattern 3 is only used more frequently at 45° and 90°.

A *X*^2^ fitting test shows that the observed frequencies of gaze patterns for the item ‘wrench’ (*X*^2^(3, *n* = 205) = 71.195, *p* = .000) and for the item brush (*X*^2^(3, *n* = 203) = 199.936, *p* = .000) differ significantly from the expected frequencies. A Kruskal-Wallis test for the item ‘wrench’ confirms that the distribution of the gaze patterns over the angles is not equal (*X*^2^(7, *n* = 205) = 14.829, *p* = .038). Following post-hoc tests (Dunn-Bonferroni tests) show that only the angles 0° and 135° tend to differ significantly with regard to the gaze patterns (*z* = -3.083, *p* = .057), effect strength according to Cohen (1992): *r* = 0.441). In contrast, the results of the Kruskal-Wallis test for the item brush show that the distribution of the gaze patterns over the angles does not differ significantly (*X*^2^(7, *n* = 203) = 7.938, *p* = .338).

Overall, the results of the different usage of the four gaze patterns for the items wrench and brush indicate that various strategies are underlying these gaze patterns which are used to solve the two items.

*Gaze pattern and performance.* With regard to performance, it can be assumed that the different gaze patterns or solution strategies lead to different success rates.

Looking at the overall success rate, it can be noted that it is over 80% for all gaze patterns for the two items. Nevertheless, there are differences between the gaze patterns in terms of error rates (Table 1). For the item wrench, gaze pattern 2 (“elaborate”) is the one with the lowest error rate (12.5%), whereas gaze pattern 4 (“uncertain”) has the highest error rate (19%). For the item brush, gaze pattern 3 (“square”) shows the lowest error rate (only 4.5%). Again, gaze pattern 4 (“uncertain”) has the highest error rate (16.7%).

**Table 1:** The percentage of correctly and incorrectly solved items for wrench and brush in the different gaze patterns.

	Wrench				Brush			
Item solved…	Gaze Pattern 1	Gaze Pattern 2	Gaze Pattern 3	Gaze Pattern 4	Gaze Pattern 1	Gaze Pattern 2	Gaze Pattern 3	Gaze Pattern 4
correct	86,6%	87,5%	86,0%	66,7%	87,4%	91,7%	95,5%	83,3%

**Figure 8:**
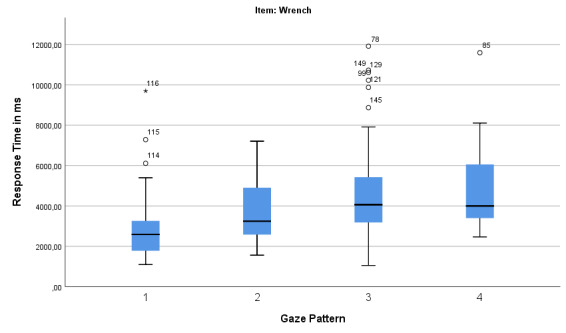
Boxplot for gaze pattern and response time (in ms) for the item wrench.

*Gaze pattern and response time for the items wrench and brush.* Differences can be observed in the relationship between the frequency of the gaze patterns and the response time. With regard to the response time, it can be stated that for the wrench (Figure 8), gaze pattern 3 (“Square”) has the largest range in response time. Gaze pattern 1 (“Analytic”), on the other hand, has the largest accumulation at a rather short response time (with the exception of three outliers).

**Figure 9:**
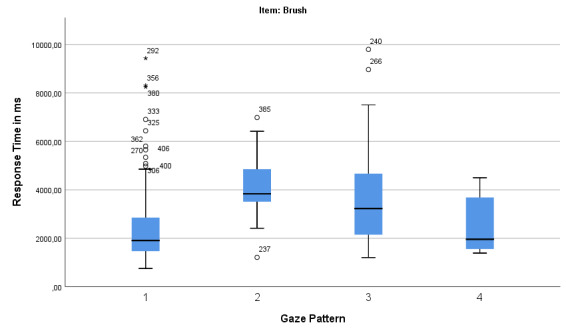
Boxplot for Gaze Pattern and Response Time (in ms) for the item brush.

In the case of the brush (Figure 9), gaze pattern 1 (“Analytic”) has the largest range of response time followed by gaze pattern 3 (“Square”). However, again, gaze pattern 1 (“Analytic”) has the greatest accumulation at a short response time.

A performed univariate ANOVA confirms differences between the response time as the within-participant factor and the gaze patterns as the between-participant factor for both items. It is shown that the gaze patterns has a significant influence with the response time for the item wrench (*F*(3,205) = 13.141, *p* = .000, *η_p_^2^* = .164). Bonferroni-corrected post-hoc tests show that for the item wrench only the gaze patterns Square (*M* = 4609.50, *SD* = 2198.72) and Uncertain (*M* = 4848.33, *SD* = 2356.59) differ significantly from the gaze pattern Analytic (*M* = 2788.36, *SD* = 1475.64).

For the item brush there are also significant differences between the gaze patterns and the response time (*F*(3,203) = 9.530, *p* = .000, *η_p_^2^* = .164). Bonferroni-corrected post-hoc tests show that for the item brush only the gaze patterns Elaborate (*M* = 4118.00, *SD* = 1566.85) and Square (*M* = 3680.64, *SD* = 2052.95) differ significantly from the gaze pattern Analytic (*M* = 2408.89, *SD* = 1517.65).

**Figure 10:**
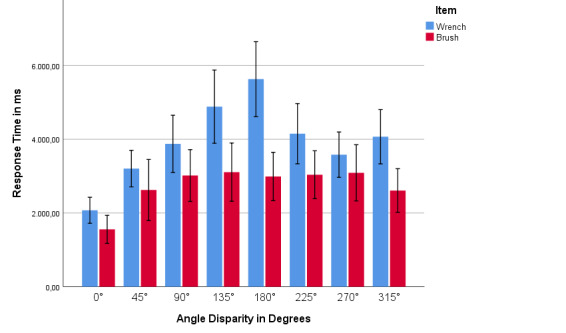
The average response time of the subjects per angle for the items wrench and brush.

*Response time and angle for wrench and brush.* We took a look at the differences between response times and angles in order to identify the underlying strategies such as egocentric and object-based [16]. To verify if there are differences between the response times and the angles, we calculated a univariate ANOVA with the response times as the within-participant factor and the angles as the between-participant factor. It is shown that the angle has a significant influence with the response time for the item wrench (*F*(7,208) = 8.438, *p* = .000, *η_p_^2^* = .228). For the item brush there is also a significant relationship between the angle and the response time (*F*(7,208) = 2.485, *p* = .018, *η_p_^2^* = .080). Table 2 shows the results of the Bonferroni-corrected post-hoc tests. Only the significant differences in response times between the angles are shown here. It is evident that there are more significant differences in response time between the angles for the wrench item than for the brush. The diagram in Figure 10 shows the average response time of the subjects per angle and item. It should be noted that the response time for the item wrench steeply increases with the angle of up to 180° and then, sharply decreases again. With the item brush, on the other hand, the increase is smaller and the curve stays flatter. According to Kaltner, Jansen and Riecke [28] such curves are characteristic for different types of items. The steeper curve is typical for an object-based mental rotation, while the flatter curve indicates a rather egocentric mental rotation. Therewith, the selected items wrench and brush seem to belong to different groups of items, also showing the features of this item group. This might be the reason why the frequency and the effectiveness of the different gaze patterns differs between the two items. The findings we revealed with our eye-tracking data analyses seem to be in line with this item and strategy classification.

**Table 2:** Results of Bonferroni corrected post-hoc tests for univariate ANOVA (angle and response time).

Item	Angle in Degrees (I)	Angle in Degrees (J)	mean Difference of response time (I-J)	Significance
Wrench	0°	90°	-1802.23	.018
		135°	-2811.62	.000
		180°	-3558.27	.000
		225°	-2077.38	.003
		315°	-1995.50	.005
	45°	135°	-1680.65	.040
		180°	-2427.31	.000
	90°	180°	-1756.04	.024
	180°	270°	2049.54	.003
Brush	0°	135°	-1551.58	.031
		270°	-1532.46	.036

## Discussion

In this study, we followed a different, rather exploratory approach using an eye tracker to record the scan paths of subjects when solving a mental rotation task with gender-stereotyped objects. We tried to analyze their gaze patterns in order to determine strategies based on them.

According to the qualitative analyses, it can be stated that according to the scan paths four gaze patterns (“Analytic”, “Elaborate”, “Square”, “Uncertain”) can be identified. More precisely, these are used by the subjects when they compare the two gender-stereotyped stimuli in one item and decide whether they are similar or different. First, we found uniform gaze patterns for gender-stereotyped stimuli. These gaze patterns were found with all angles and with both items, namely wrench and brush. Furthermore, it can be observed that the frequency of the four gaze patterns differs between the items: gaze pattern 3 (“Square”) is most frequent in the item wrench, while gaze pattern 1 (“Analytic”) dominates in the brush. Interestingly, the dominant gaze patterns do not show the lowest error rates. For the item wrench, gaze pattern 2 (“Elaborate”) has the lowest error rate (12.5%), while gaze pattern 3 (“Square”) has the lowest error rate for the item brush. Thus, there appears to be no direct relationship between the frequency of the gaze patterns and the performance. However, the sample was too small for more detailed analysis and further studies should examine this.

With regard to the question of whether solution strategies can be derived from the gaze patterns, we found some hints in our results. If gaze patterns are preferred with an item and if the gaze patterns correlate differently with performance, they can be interpreted as an indicator for various solution strategies. This is in line with the finding that there is a relationship between gaze pattern, response time and solution strategies [e.g. 29, 30], i.e. some gaze patterns, and solution strategies respectively, seem to be faster than others. Khooshabeh et. al [30] state in their results that "good imagers were less accurate and had longer response times on fragmented figures than on complete figures. Poor imagers performed similarly on fragmented and complete figures. These results suggest that good imagers use holistic mental rotation strategies by default, but switch to alternative strategies depending on task demands, whereas poor imagers are less flexible and use piecemeal strategies regardless of the task demands." In terms of our results, this means that depending on the angular disparity and difficulty of the item to be solved, accuracy decreases and response times get longer. Thus, response times for the wrench item increase linearly with angular disparity. This suggests a dynamic imaging process that resembles actual physical rotation and for which mental rotation has been assumed to be based on visual representation. This theoretical approach, in turn, is known as holistic [31, 32]. However, more detailed analyses of a supposed strategy require a larger sample. This should be done in further studies. Furthermore, the gaze patterns in our experiment are not congruent across the subjects, which indicates a change in strategy depending on the level of difficulty.

So, we developed and described a new approach for a bottom-up identification of the strategies, the subjects use in a mental rotation task. We described how the analysis of the gaze patterns works and how informative such an approach can be for the two example items wrench and brush. Furthermore, we found evidence that our findings correlate with the literature on mental-rotation strategies and on different item types for mental rotation respectively. While the brush seems to rather induce an egocentric mental rotation, according to its angle-response time curve, the wrench appears to evoke a rather object-based mental rotation. This seems to be in line with the findings on dominance and successfulness of the gaze patterns we identified for the two items. Hence, we can assume that the gaze patterns and the solution strategies underlying them are congruent with the mental-rotation strategies described in the literature.

Future studies including more and different items than those used in this study as well as studies with larger sample sizes are necessary. Therewith, the developed method should further be evaluated and more information on the strategies underlying the identified gaze pattern should be acquainted. In regard to the fact that in mental rotation tasks the strategies described in the literature seem to differ according to gender [e.g. 33, 20, 24]. So in future studies with a larger number of subjects should be investigated whether the gaze patterns we found also differ in terms of frequency among the sexes.

As we have already noticed, for future studies on mental rotation and eye tracking with gender-stereotyped items, it is necessary to use equally difficult stimuli resp. objects. The eye tracking data and the behavioral data showed that the brush was easier to solve than the wrench. This might have had an influence on the gaze patterns of the subjects. Our results also outline that the gaze pattern, the underlying strategy and the performance on an item depends on the visual characteristics of the object and the angle in which it is rotated. Therefore, according to the visual features, the selection of the stimuli appears to be an important issue for future mental-rotation research, especially when using eye-tracking as a method.

### Ethics and Conflict of Interest

The author(s) declare(s) that the contents of the article are in agreement with the ethics described in http://biblio.unibe.ch/portale/elibrary/BOP/jemr/ethics.html and that there is no conflict of interest regarding the publication of this paper.
